# Medical assistance in dying and the meaning of care: Perspectives of nurses, pharmacists, and social workers

**DOI:** 10.1177/1363459321996774

**Published:** 2021-03-08

**Authors:** Anneliese Mills, Kristin Bright, Rachel Wortzman, Sally Bean, Debbie Selby

**Affiliations:** University of Toronto, Canada; University of Toronto, Canada; Middlebury College, USA; University of Toronto, Canada; Sunnybrook Health Sciences Center, Canada; Department of Family and Community Medicine, Canada; Sunnybrook Health Sciences Center, Canada; University of Toronto, Canada; University of Toronto, Canada; Sunnybrook Health Sciences Center, Canada; Department of Family and Community Medicine, Canada

**Keywords:** Medical Assistance in Dying, care, nursing, pharmacy, social work

## Abstract

Medical Assistance in Dying (MAiD) was legalized in Canada in 2016. While it has generated significant academic interest, the experiences of healthcare workers other than physicians remain understudied. This paper reports on a qualitative study of interprofessional Healthcare Providers (HCPs) involved in the provision of MAiD in order to: (1) characterize providers’ views about the care they offer in general; (2) examine whether or not they consider MAiD a form of care; and (3) explore their reasons for viewing or not viewing MAiD as care. Semi-structured qualitative interviews were conducted with ten nurses, eight social workers, and three pharmacists with firsthand experience delivering MAiD at an academic hospital in Toronto, Canada. The study was approved by the hospital’s REB. Written informed consent was obtained prior to participation. Codebook thematic analysis and template analysis generated four themes: (1) care as advocacy, (2) care as easing suffering, (3) care as psychosocial, and (4) care as relational. Every participant viewed MAiD as a form of care and drew on these four themes to authenticate MAiD as care. Participants consider MAiD a form of care for patients, families, other healthcare workers, and even themselves. In alternating and composite fashion, they describe MAiD in terms of autonomy, easing suffering, and a kind death for the dying (and those entrusted with their care)—a complex choreography of social discourses and moral logics that refuse to settle into a simple dichotomy of “choice versus care.” Participants depict MAiD in many of the same terms and imagery they use to describe the care they offer in general. In light of ongoing social controversies surrounding MAiD, HCPs utilize a range of logics strategically to repel negative attention and enable their participation in what they see as a caring end for their patients.

## Introduction

Physician assistance with dying, variably termed voluntary euthanasia, assisted suicide, or Medical Assistance in Dying (MAiD), has become increasingly available in multiple countries (Dyer, 2008; Orentlicher, 2016; Watson, 2009). Canada legalized such assistance through the passage of Bill C-14 in 2016, which amended the federal Criminal Code allowing physicians and nurse practitioners to provide MAiD to patients meeting specific criteria (Carter v. Canada 2015; Criminal Code, 1985). MAiD has generated growing academic interest, particularly in areas of program implementation and patient data (Ball et al., 2018; Downar et al., 2020; Li et al., 2017; Selby et al., 2020; Wales et al., 2018; Wiebe et al., 2018). There is active debate around topics such as ethics, barriers to access, and the ability of physicians to act as “conscientious objectors” in light of unclear professional obligations to provide “effective referral” (Heilman and Trothen, 2020; Sikka, 2019; Will, 2019; Wright and Shaw, 2019; Zolf, 2019). However, researchers have almost exclusively considered patients or physicians, with only a handful of studies examining interprofessional Healthcare Providers (HCPs). This is indicative of a broader trend in social scientific studies where qualitative researchers tend to focus on the patient-physician dyad rather than the interprofessional healthcare team.

Fujioka et al. (2018) published a scoping review of providers’ perspectives of MAiD and highlighted the need for more research, particularly among interprofessional HCPs. Brooks (2019) authored an additional review, confirming that the vast majority of articles published internationally on assisted death focused on physicians. Likewise, Suva et al.’s (2019) review called for more research on MAiD and nursing. Several teams have considered HCPs and MAiD, although many are editorials or case studies (Antifaeff, 2019; Banner et al., 2019; Beuthin and Bruce, 2018; Pesut et al., 2019; Schiller et al., 2019; Thiele and Dunsford, 2017). MAiD constitutes a major paradigm change in end-of-life care that builds upon longstanding debates while opening up new questions about death and dying, palliation, ethics, and the roles of MAiD professionals. Despite these shifts, a majority of studies of HCPs, where those collect data at all, are informed by quantitative measures (Antonacci et al., 2019; Freeman et al., 2019; Gallagher et al., 2019; Murphy et al., 2018; Verweel et al., 2018) with few employing qualitative methods (Beuthin et al., 2018; Brown et al., 2020; Bruce and Beuthin, 2019; Pesut et al., 2020). The paucity of qualitative data suggests that new configurations of meaning, discourse, and debate accompanying MAiD are underexplored.

This article reports on an in-depth qualitative study of HCP perceptions of MAiD and care. We interviewed nurses, social workers, and pharmacists who had firsthand experience with patients considering or receiving MAiD. In brief, our study aimed to: (1) characterize how HCPs perceive their roles as care providers; (2) examine whether or not they view MAiD as a form of care; and (3) explore their reasons for viewing or not viewing MAiD as care.

To contextualize these questions, we provide a brief history of MAiD and its legalization. Then we map out our interview methodology and present four thematic findings with respect to HCP perspectives on care: (1) care as advocacy; (2) care as easing suffering; (3) care as psychosocial; and (4) care as relational. We then analyze HCP views on MAiD, where we found salience for the same four themes. Lastly, we interface our analysis of HCP perspectives with critical feminist science studies and the anthropology of care, particularly Mol (2008), Kaufman (2005), and Livingston (2012). With regard to Mol’s critique of a dichotomy of “choice” and “care” under industrialized biomedicine, participants supported both logics as significant to MAiD while expanding on the meaning of each rationale. We conclude that, in light of ongoing controversies surrounding MAiD, HCPs strategically use a range of meanings of care to resist scrutiny and enable their participation in what they see as a caring end for their patients. These qualitative meanings have particular import for multidisciplinary settings where not all team members or professionals support MAiD.

## Background: MAiD and the “right to die” movement

Medical Assistance in Dying came about as a result of a *Charter* Challenge—the Supreme Court of Canada ruled that the criminal prohibition on physician-assisted suicide violated the rights of Canadians with severe and life-limiting illnesses. In this sense, MAiD arose through litigious and rights-based mechanisms. The eligibility criteria likewise support the notion of individual autonomy in the setting of suffering. In order to access MAiD, an individual must:

- be at least 18 years old and be capable of making health care decisions.
- have a grievous and irremediable medical condition (defined briefly as an advanced illness causing intolerable suffering to the patient, in the setting of a reasonably foreseeable natural death).
- make a voluntary request for medical assistance in dying that is not the result of outside pressure or influence.
- give informed consent (Criminal Code; GC (Government of Canada), 2018).

These criteria paint a picture akin to Mol’s (2008) “logic of choice,” a system in which patients are treated as autonomous consumers or citizens capable of making their own decisions if they are provided with impartial information. Scholars juxtapose choice not just with “care” (as Mol does), but also “trust” and “shared decision-making” (Downie, 1998; O’Neill, 2002; Sandman and Munthe, 2010). These studies are part of a broader critique of choice-based healthcare decision-making as an artifact of neoliberal capitalism (e.g. Ayo, 2012; Gott et al., 2008; Henwood et al., 2011; Petryna, 2009). Importantly, scholars also highlight a dichotomy between patient agency and the authority of physicians (i.e. paternalism). These studies thus locate choice and agency within the patient-doctor relationship and, consequently, obscure the role of interprofessional HCPs.

MAiD is often compared to earlier forms of hospitalized death such as withdrawal of care, palliative sedation, or progression of an illness to its natural end. Some scholars have questioned the ethical distinctions between these scenarios (Lipuma, 2013; Ten Have and Welie, 2014; Wright and Shaw, 2019) and there is active debate (Booker and Bruce, 2020). However, there are several key differences worth highlighting. One of the critical distinctions is that MAiD is guided by the “capable patient,” whereas other kinds of death may involve patients who are delirious or unconscious, with strong family involvement (Crowley-Matoka, 2016; Warraich, 2017). Decisions about death in these cases are frequently made by the family, with varying levels of input from the semi-conscious patient or their advance care plan (Warraich; Crowley-Matoka; Lock, 2000). Death itself can take days or weeks yet may still be perceived as “unexpected” (Russ and Kaufman, 2005). In certain ways, this is the opposite of MAiD, where the time of death is known in advance, almost to the minute. On a spectrum of choice to care, MAiD is uniquely seen as a “choice” in law, policy, and ethics. It allows a death for patients who are suffering that is premised on individual rights and full rationality. However, this vision is based in legal precedent and individualistic logics (e.g. informed consent). In the following sections, we present the methods, results, and analyses we executed to explore how HCPs view the profoundly social situation of MAiD.

## Methods

This study consisted of semi-structured interviews conducted with nurses, pharmacists, and social workers at a tertiary hospital in Toronto, Canada. Eligibility criteria required direct professional experience with MAiD, defined as involvement when a patient was considering, requesting, or receiving MAiD. Recruitment occurred via email invitations sent by professional practice leaders, with those interested contacting the study coordinators. The participants included 10 nurses (including patient care managers), 8 social workers, and 3 pharmacists, all from multiple departments in the hospital. Interviews occurred between May and July of 2019 and were approximately 30–60 minutes in length. The interview guide is attached as Appendix A. Data collection and analysis occurred simultaneously (Merriam and Tisdell, 2015) which allowed the interviewer to explore salient ideas raised in previous interviews. Participants were asked for permission to record their interviews, which were subsequently transcribed verbatim. Audio files were deleted upon transcription and transcripts were anonymized by omitting any reference to participants, other personnel, or specific hospital units. The project was approved by the hospital’s institutional REB. Every participant provided written informed consent prior to being interviewed.

The research team consisted of a Master’s student in anthropology, a cultural medical anthropologist, two palliative care physicians, and a clinical ethicist. The latter three investigators had firsthand experience with different stages of the MAiD process, from initial requests to ultimate provision. In order to minimize this bias, all of the interviews were conducted by the Master’s student, who had previously contributed to qualitative, interview-based projects. Data analysis was inductive (Bradley et al., 2007). Specifically, our framework closely resembled “codebook thematic analysis” (Braun and Clarke, 2019; Braun et al., 2018) or “template analysis” (Brooks et al., 2015) in that the main technique was line-by-line coding according to an overarching coding structure. However, most of the coding occurred during the interview period, enabling an iterative approach (Charmaz and Belgrave, 2012; Lingard et al., 2008). The coding structure was jointly developed by three investigators after they had familiarized themselves with the first six interviews and the structure was refined periodically throughout the remainder of the study. These three investigators coded the same four interviews; subsequently, every interview was coded by the Master’s student and at least one other investigator. Themes were generated, revised, and defined by the entire research team in a collaborative fashion. This article reports specifically on our thematic results pertaining to care.

## Results

Interviewer: *. . . What is your role in offering care? As you see it.*

Social Worker: *As I see it. Being with them. Listening with them. Understanding what’s important to them. Sometimes care has nothing to do with the medical care. It’s about other things. Understanding their goals, helping them reach their goals. Helping them be realistic about the healthcare system, what we can and cannot provide. It’s advocating for patients and families and getting resources. Meeting them where they’re at and exploring with them what they need at that moment, at that time. Bringing humour into this too [. . .]. I can remember for one of our patients [. . .]he was joking and laughing while he was talking about his final days. He ended up having a glass of champagne with his sister before his procedure. Another patient, an ALS patient, they wanted ice cream so there was a gelato party in the room before the procedure. That’s caring. It’s not always about the MRIs and the surgeries and the chemo. It’s about listening and it’s about gelato ice cream. That’s caring, how I see it.*

Participants were asked how they felt they offered care in the course of their ordinary working life—that is, not necessarily in the context of MAiD. Every participant endorsed providing some kind of care, with the exception of one social worker who preferred the term “support.” During analysis, “care” was designated a major code which subsequently generated four themes.

### Theme 1: Care as advocacy

Advocacy was frequently cited as a key component of care. There were consistently two levels of advocacy. The first was between the patient and the interprofessional healthcare team. This seldom took the form of formal lobbying, but rather more subtle efforts, usually to help the team understand why a patient might want a course of action or why that desire would need to be respected. The second level was to advocate on behalf of the patient within that patient’s family or support network. Social workers, in particular, often worked extensively to help families understand or accept a patient’s treatment decision or advance care plan. In general, advocacy was linked to patient autonomy in decision-making. Here are typical examples:

*I can obviously advocate to the physician on behalf of that patient. . . patients say a lot of things to us nurses.*

*I think one of the biggest roles we can be as nurses are advocates, and advocating for what your patient wants isn’t necessarily what you would want for yourself. I think that’s something very important to remember. [. . .] So I think part of caring for your patient is really carrying out their directives for themselves too.*

*I think [social workers] hold a lot of valuable information, and being able to speak on behalf of the patient, or helping [the healthcare team] understand why a patient’s coming to a decision has been very helpful for our nursing staff or other staff.*

*A huge core value of social work is the autonomy and the advocacy. I think probably more than anything, it’s just giving our patients every and all option that’s available to them that would be appropriate in their particular circumstance.*

### Theme 2: Care as easing suffering

Suffering was a key, and recurring, theme in the nursing interviews. Specifically easing suffering, or ensuring comfort, was discussed prominently by every nurse with clinical duties. In this sense, nurses often viewed MAiD as an extension of their usual care. Here are typical answers nurses gave when asked to describe their usual professional care:

*My caring is [. . .] making them as comfortable as possible, ease their suffering. Whether it’s giving them pain medication or helping them sleep or decreasing their nausea to make them more comfortable. . . goes right along with MAiD where their suffering is so immense that they don’t want anything else to happen. I think it’s all part of the same idea: end their suffering.*

*Where I work, the area I work, I would say if I can make the transition for them more comfortable. . . then I know I’ve done a good job.*

*I’m there as a palliative care nurse to provide as much comfort as possible. This is part of my training or my philosophy that I am providing the ultimate comfort for these people. They don’t have to be in pain, or they don’t have to be frustrated. They don’t have to worry about losing their faculties. . .*

As can be seen by the last quotation, there was sometimes considerable overlap between physical, psychological, and social suffering. However, biomedical understandings of suffering were uniquely important for these nurses in the sense of comfort and symptom management.

None of the social workers in this study specifically drew on suffering or comfort in describing the kind of care they offer to patients in general. However, the concept of suffering was relevant for social workers in the sense of bearing witness, or contextualizing, patients’ psychosocial suffering. This is discussed further in Theme 3.

### Theme 3: Care as psychosocial

Participants from all professions repeatedly wove psychosocial aspects of care into the interviews, including emotions, spirituality, coping, and empathy. Social workers often explicitly referred to these dynamics as “psychosocial” and depicted them as the mainstay of their care. For other HCPs, these aspects often followed the more practical or technical aspects of their job, but were usually given equal weight. For example, one pharmacist began by depicting their care in terms of their role as a “drug expert,” but most of the answer centered on this point:

*But then overall, being empathetic to any other needs that come up and showing compassion and understanding. Even if it’s something that might not be medication-related. And that often happens a lot when you go in to speak to someone about their meds. They’ll be, they’ll have another issue, that is not really a pharmacy issue, but being able to empathize with them. Like ‘oh, I’m sorry, I’m really sorry. It’s a really crappy place to sleep, you can’t get any sleep, I understand. Let’s talk to the nursing staff to see if we can do something to help with that.’ So also recognizing that you might be doing things outside of your trained role that will help the patient.*

This sentiment is consistent with Biehl’s (2007) distinction between care and pharmaceuticalization; the pharmacist saw titrating medications as only part of genuine care.

Explanations of social and psychological care were prevalent in the interviews with social workers. Within this theme, important concepts included the creation of a safe space and bearing witness to suffering, as evidenced by these social workers:

*Care to me means physical, emotional, psychological and spiritual provision of support. That’s how I see my role. . . How is the patient coping emotionally, and what can I do to provide support to them?*

*Care for my patients is providing a safe space for them to explore their suffering, for me to be able to bear witness to their sadness without trying to fix or pathologize what their experience is. There’s a place for them to hold their suffering, where others in their family or in their community are not able to hold that. They’re always trying to fix the sadness or the suffering. I think part of my role as a social worker in caring for them is to be able to bear witness to their suffering and to help them put their life into context, which would elicit emotion. To be able for them to form a narrative around their life, and to talk about what their main purpose in life has been, and what their legacy is.*

Many of the social workers linked their acknowledgment of pain to their role in MAiD. As friends and family may try to mollify a patient’s experience of pain, social workers are sometimes the first people patients encounter who take their consideration of MAiD seriously.

### Theme 4: Care as relational

A recurring theme in every interview was that care is relational. Among the three most salient concepts, the first was the relation of patient and HCP. This was a strong theme amongst the pharmacists; two of them described this as the main form of their care.

*I learned very quickly you can have your agenda but you’ve got to be very fluid. If they want a more paternalistic approach and they want to have it all broken down; if they want to have a very fluid dialogue because they’ve done research; if they want to talk about their dog or their tumour, then I think that is as important – if not more – than dispensing of clinical knowledge. It means establishing a relationship, a rapport which then will make them more open to hearing info as you come.*

The next and most significant locus of relational care was the family. The vast majority of HCPs in this study explicitly volunteered that part of their role was caring for the family. A social worker offered the following comment spontaneously:

*Caring for the patient is caring for the family. A big part of my work is for the family to be able to explore what the patient – how their life will continue when they are no longer here. That’s the crux of my care for the patient and family.*

Social workers in particular offered tangible services to families, including meeting with them and helping with funeral planning after a patient’s death. In this way, social workers viewed care for an individual patient as encompassing care for their extended social network.

The final level of relational care was not present in all, or even a majority of interviews, but is notable for its inclusion in a specific group that has thus far been underexplored. The patient care managers were clear that they viewed themselves as offering care, despite their administrative and managerial role. They articulated that they cared for other HCPs, and in so doing, also cared for patients:

*Yeah, I would see it as offering care. Which is a bit odd because I don’t have any direct contact with the patient or the family. I’m doing my piece in terms of helping them with what they want.*

*Care for me is about . . . Care for the staff; care for the patients; care for the families; care for running a unit in a caring way that reflects staff caring for patient and families. All staff: support staff, allied staff, health professions. [. . .] If you care for your staff, and they feel cared for, everything else falls into place. The outcomes are better for patients. The caring is better for patients.*

This idea was occasionally discussed by other HCPs, but never as saliently as for the patient care managers. Overall, it is clear from this theme that HCPs conceive of care as relational by creating relationships with their patients directly, by supporting patients’ families, and by supporting other staff members within the circle of care.

## MAiD as care?

Nurse: *MAiD must be a kind of care. . . you must be a very caring person before you can be a MAiD nurse.*

Participants were asked if they ever had any difficulty reconciling their views of care with their role in MAiD. Every HCP without exception responded that they did not have difficulty and confirmed that they view MAiD as offering care. The nurse quoted above was the only one who did so after any amount of hesitation. The other participants in this study responded immediately and enthusiastically. Multiple people replied with “absolutely” or “100%.”

The reasons offered to justify this position were in keeping with the four themes discussed previously—that is, advocacy, easing suffering, psychosocial care, and relational care. In terms of advocacy, nurses and social workers felt that they offered care when they advocated for the choices, including MAiD, that patients made with their own autonomy. Several participants appealed to the notion that MAiD was a means of ending suffering; this was true even for social workers who had not mentioned suffering or comfort when describing their usual care. These two reasons—advocacy/autonomy and easing suffering—were typically the first reasons participants listed. Later in the interview, HCPs tended to discuss a number of psychosocial and relational elements of MAiD, including that patients and families have time to prepare for a person’s death and that the patient would die while still “being themselves.” All of the participants felt that MAiD offered care to families as well as patients. A select number also mentioned caring for other HCPs. For instance, one nurse reported that, in addition to other reasons, they would always assist with MAiD because of “support for the other staff member in there too”—namely, that without their participation, the physician would be in the room on their own.

One of the most evocative parts of the interview took place when participants explained why they felt MAiD was a more caring form of death. This was particularly true for nurses, who offered the following comments:

*They have a nice evening before their MAiD, they have their music, a little food, friends come over. The patients are able to communicate. . . Other times when people come, the person is unable to communicate. Their level of consciousness is very poor, and people don’t want to see them like that.*

*The process is so incredibly smooth and peaceful that there’s no trauma involved in watching it happen. [. . .] The patient literally says yeah, I feel . . . and they’re asleep. And then the stuff that ends their life happens while they’re sound asleep. It’s really incredibly peaceful. I think the whole thing takes something like five minutes, and they’re asleep after the first 30 seconds.*

*The positive thing about it is, you know when, you know who’s going to be present. Sometimes when people are dying we can call the family in, but they don’t always make it. Plus you avoid some of those symptoms. They’re no good. There’s no gasping for air. The people I’ve seen, it looks like they go to sleep. It’s very peaceful.*

*I feel like it almost gives [the family] control over the situation. They’re able to go, OK, it’s going to be on this day, we’re going to have these people present, we’re going to listen to this music, we’re going to bring this special blanket in, we’re going to have this food, or something like that. Then they can kind of create their situation, which I think is very unique and beautiful. . . I think MAiD kind of helps people prepare a little bit better.*

The participants quoted above depicted MAiD as a kind death. This tended to be for several reasons: (1) the death itself is quick and peaceful; (2) pursuing MAiD allows patients to bypass unpleasant end-of-life symptoms; and (3) MAiD gives families an opportunity to accept the death, prepare accordingly, and say goodbye. By framing MAiD as kind in this way, participants revealed a further layer of relational care. These comments suggest that, in addition to caring for patients, families, and other staff, MAiD can offer care to providers themselves. This is obvious not only in their positive terminology (“peaceful” and “beautiful”), but also in their negative descriptions of other deaths.

The question of MAiD as a form of care elicited some of the most glowing affirmations of the practice. Even though participants were not asked about their own end-of-life wishes, two nurses volunteered that they would personally like to die by MAiD. An additional nurse discussed a family member who had passed away prior to the legalization of MAiD:

*I think if he could have had [MAiD], I think it would have been better for him. I think it would have been better for everyone. Than to watch someone just slowly lose everything. And not have to make decisions like do you put in a feeding tube? When you know someone’s dying, but you know they’re mentally there, those decisions that you probably could have just avoided.*

It was clear from this section of the interview that participants see MAiD as more compassionate than dying by other means. Specifically, they described MAiD as kinder to patients in terms of physical, psychological, and social suffering; they likewise described it as better for families.

## Discussion

Critical health studies have often produced a coherent vision of care as being social, temporal, and relational, as opposed to predicated on autonomy and choice. In addition to Mol, several ethnographers working in the end-of-life sphere are critical of choice or, alternately, the façade of choice (Ayo, 2012; Ellis et al, 2017; Gott et al., 2008; Henwood et al., 2011; Petryna, 2009). Kaufman (2005) researched hospitalized dying and described the ways families are forced to make intractable decisions, while death is prolonged and patients reside in the “gray zone of indistinction.” She discusses the ways family members are placed in perverse situations where they have to make incredibly difficult decisions, the medical realities of which are beyond their comprehension. This continues even when the healthcare team feels as though there is no hope for recovery due to the “imperative to treat” (Chapple, 2003; Kaufman, 2005; Kaufman et al., 2004; Lawton, 2000; Russ and Kaufman, 2005). This imperative is similar to Stevenson’s (2014) concept of “biopolitical care,” where institutions become concerned purely with the “maintenance of life itself,” rather than caring for patients as individuals. An important aspect of Kaufman’s work is temporality, as hospital dying can be prolonged almost indefinitely. One ethnographer who depicts a caring scenario is Livingston (2012), who describes nurses in Botswana participating in social healing, particularly through laughter and empathy. Her description is consistent with Mol’s dichotomy in that choice, at least in the consumerist sense, is almost completely absent in the oncology ward she details.

In this discussion, we consider how HCPs draw on discourses of care and choice in their discussion of MAiD and their role in its delivery. Two coexisting ideas appear in the way HCPs view their care, including MAiD. On the one hand, participants frequently invoked choice-based reasoning: they are advocates, ensuring that other healthcare workers respect patients’ autonomy and enact their decisions. Correspondingly, they viewed MAiD as care because of the control, autonomy, and empowerment it offers patients. On the other hand, many HCPs framed their care in terms of alleviating suffering, both symptomatically and psychosocially. In these terms, MAiD becomes the ultimate way of relieving suffering—partly because of its finality, but also because of the ability it gives patients and families to plan and prepare (e.g. by congregating and planning the patient’s final moments). This second rationale includes both relational and temporal aspects, and is reminiscent of the care depicted by the aforementioned scholars.

The relational aspects were striking. For example, one participant’s comment that “caring for the patient is caring for the family” endorses a certain kind of “mutuality of being,” a phrase of Sahlins’ (2013) that has been used in the context of cancer care (Bright, 2015). Additionally, MAiD is truly the option many participants would prefer for their patients. This bears a close resemblance to Mol’s “logic of care.” HCPs are not simply impartial purveyors of healthcare goods; they are invested parties that are relieved to see patients bypass horrible end-of-life symptoms and families forego weeks of uncertainty in Kaufman’s “gray zone.” In fact, MAiD might be the most caring option for certain HCPs themselves, as it may relieve the distress they experience in caring for patients with significant suffering (and, in this way, satisfy the ubiquitous desire to “move things along” (Kaufman, 2005)).

In contrast with the dichotomization of choice and care articulated by scholars (Downie, 1998; Mol, 2008; O’Neill, 2002; Sandman and Munthe 2010), the HCPs in this study articulated and relied on a composite. Other researchers have observed situations that defy or exceed this dichotomy, such as Bright (2015), who writes about patients seeking breast cancer treatment in India, and Yates-Doerr (2012), who presents the experiences of individuals seeking care at a Guatemalan dietary clinic. Likewise, Borgstrom and Walter (2015) discussed English end-of-life policy and the idea that choice and compassionate care might 1 day “cooperate as friends.” However, it is interesting to analyse this dynamic further. What is striking about the data at hand is the pattern through which these logics were used: participants usually espoused a choice-based idea of care (i.e. advocacy and autonomy) early in the interview, then shifted to more relational accounts, employing lengthier explanations and a greater sense of intimacy and imagery.

It is difficult to understand this pattern without considering participants’ work settings. In another paper, we explore the unique interpersonal situations HCPs face (Mills et al., 2020). Several participants described units where the culture was strongly opposed to MAiD. They explained that they did not always feel comfortable expressing their opinions about MAiD and could not have productive conversations about it with a majority of colleagues. It is perhaps unsurprising then that HCPs appeal to logics of patient autonomy by describing their role as enacting a *patient’s* wishes. This does not mean that practitioners’ sentiments about patient choice were inauthentic; however, foregrounding that aspect of care may deflect unwanted attention and opposition. In this situation, then, a logic of choice is used not as one position, but a composition of braided logics including patient autonomy, decisional agency, avoidance of negative or retaliatory perceptions of colleagues, and advocacy for MAiD as an end to suffering.

On one hand, choice is *not* care-based, tracking with Mol’s dichotomy. On the other hand, choice plays a crucial role in this scenario—that is, participation in a contentious end-of-life paradigm—by allowing HCPs to contribute to MAiD while minimizing scrutiny from their colleagues. In a political landscape dominated by “effective referral” and “conscientious objection,” it can be difficult for patients to find staff willing to enact MAiD. In this sense, comments on respecting patient’s wishes irrespective of personal values do not simply coexist with care, they enable care. They have particular utility from an interpersonal perspective. This directly relates to the process through which MAiD was originally instituted: advocacy groups had to frame the issue as a human right in a court of law. As Ari Gandsman (2017) has written about such activists, “Individual autonomy and self-determination are necessary rhetoric but fictitious.” As was evident in this study, participants believed that MAiD offers care, at least to a subset of patients and families; nevertheless, this care would not be possible without MAiD’s deeply litigious, choice-based roots.

## Conclusion

Nurse: *Even though it’s so tough, at the end of the day, I go to bed knowing that person’s suffering is done, and I think that gives me peace. Especially because it’s what they want as well. It’s a hard profession in general.*

This project has explored the perspectives of a distinct group of people that has thus far been underrepresented in academic literature: HCPs who contribute to cases where patients consider, request, or receive Medical Assistance in Dying. It attempted to remedy the scarcity of qualitative research by conducting semi-structured interviews and employing inductive methodology. The aims were to explore how HCPs characterized their ordinary professional care, as well as whether or not they viewed MAiD as care and their justification for this stance.

With respect to participants’ care in general, we highlighted four central themes: (1) care as advocacy; (2) care as easing suffering; (3) care as psychosocial; and (4) care as relational. These themes resonated with participants’ perceptions of the care they offer through MAiD. Many participants perceived their participation in MAiD as care because they were supporting patient autonomy, while others described MAiD as palliation. Most participants articulated *both* logics at varying points in the interview. Similarly, participants repeatedly discussed MAiD as a better form of death—one that is kinder for patients and relatives. As such, for these HCPs, choice and care appear to coexist. Specifically, the logic of choice may function as a strategy to assuage vocal opposition to MAiD (Mills et al., 2020). Overall, MAiD may be a situation in which these logics are not fundamentally opposed, nor do they simply coexist, but where they enable one another to produce a caring end for patients and families.

## Limitations

This study had a small sample size, particularly with respect to pharmacists, and all of the participants self-referred, meaning they may not be representative of the broader group of HCPs participating in MAiD. Additionally, the study was based at a single tertiary hospital. The culture of MAiD provision may vary considerably between institutions and jurisdictions, which may further affect and nuance providers’ interpretation of “care.”

## Appendix A: Semi-Structured Interview Guide

Interview Questions, to be integrated when appropriate: (with follow-up prompts as needed)

1. Can you tell me about where you work in the hospital and what your role is?
2. Can you tell me about any professional experience you have had with patients considering or receiving Medical Assistance in Dying (MAiD)?
3. How did you come to be involved in the care of these patients?
4. Thinking back on your experience(s), was there anything in particular that made it especially rewarding or especially challenging?
5. On a day when you have some involvement with MAiD, how does this experience differ from your regular professional activities?
6. **A)** All of the professions interviewed for this study are often called “caring professions.” As a (nurse/pharmacist/social worker), what does care mean to you? What does care mean in the context of a seriously ill or dying patient? **B)** Have you ever had any difficulty reconciling MAiD with this understanding of care?
7. How do you think the majority of your profession feels about MAiD? Within your profession, how are people who contribute to MAiD treated? How are those who do not wish to participate treated?
8. Do you think there is enough information provided to people in your profession about MAiD? Are there adequate resources available for talking, debriefing, etc.?
9. Do you think that any of your past personal or professional experiences with death/dying have influenced the way you feel about MAiD?
10. MAiD is a new topic that has been receiving attention in terms of research, news stories, etc. Do you think that the role of your profession has been adequately represented in this literature?
11. Is there anything that could be changed to make the experience of performing this role easier or more fulfilling?
12. Is there anything else you would like to tell me about MAiD or the role of your profession?
